# Epileptic Seizure after Use of Moxifloxacin in Man with *Legionella longbeachae* Pneumonia

**DOI:** 10.3201/eid2611.191815

**Published:** 2020-11

**Authors:** Jin-Yong Wang, Xing Li, Jian-Yong Chen, Bo Tong

**Affiliations:** Department of Internal Medicine, Jiangxi Provincial People’s Hospital Affiliated to Nanchang University, Nanchang, Jiangxi, China.

**Keywords:** Legionella longbeachae, metagenomic next-generation sequencing, epileptic seizure, moxifloxacin, pneumonia, bacteria, China

## Abstract

Legionellosis caused by *Legionella longbeachae* is diagnosed mainly by PCR. We report a case of *L. longbeachae* infection in mainland China, which was diagnosed by metagenomic next-generation sequencing, in a man who developed an epileptic seizure after using moxifloxacin. Metagenomic next-generation sequencing may be a useful tool to detect *Legionella* spp.

We describe a case of *Legionella longbeachae* infection in mainland China that was diagnosed by metagenomic next-generation sequencing (mNGS). The patient developed an epileptic seizure after he underwent treatment with moxifloxacin and had a prolonged corrected QT interval at a later stage of treatment.

## Case Report

In June 2019, a 56-year-old man came to the pneumonia department of People’s Hospital Affiliated to Nanchang University with a 2-day history of nonproductive cough and fever. He had undergone a deceased-donor kidney transplant 19 years earlier for end-stage renal disease and had received regular hemodialysis for the previous 2 years. His medications included tacrolimus capsule (1.0 mg 2×/d), mycophenolate capsule (0.25 mg 2×/d), and prednisone (10 mg/d). He had recently done gardening activities, using potting mixes. On physical examination, his temperature was 39.6°C, heart rate 95 beats/min, oxygen saturation 96% while breathing ambient air. Pulmonary auscultation revealed wet rales in the right lung. The rest of the examination results were unremarkable.

**Figure 1:**
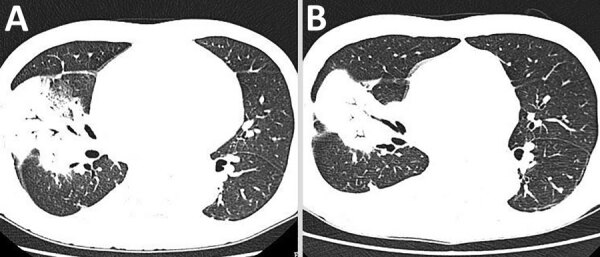
Computed tomographic scan of the chest of a patient hospitalized with *Legionella longbeachae*. A) On day 14 of the patient’s hospital stay, extensive consolidation was present in the right upper and middle lobe. B) On day 25 of the patient’s hospital stay, the consolidation was smaller than before.

The patient’s leukocyte count was 10.54 × 10^9^ cells/L (reference range 4–10×10^9^ cells/L), with 95% neutrophils; his C-reactive protein (CRP) level was 200 mg/L (reference range 0–15 mg/L). Computed tomography (CT) of the chest revealed extensive consolidation in the right upper and middle lobes (Figure 1, panel A). 

We treated the patient with 5 g of intravenous piperacillin every 12 hours. However, the patient’s symptoms did not improve. Cultures of bronchoalveolar lavage fluid (BALF) samples were negative. Results of a T-SPOT.TB test (Oxford Immunotec, https://www.tspot.com) were negative. We began intravenous meropenem (2,000 mg/d) and withdrew piperacillin. The patient’s temperature decreased but was still elevated. We performed another BAL 8 days later; cultures were still negative.

We sent a BALF sample to BGI-Wuhan (formerly Beijing Genomic Institute; Wuhan, China) for pathogenic detection by mNGS. Qualified libraries were sequenced by BGI-Wuhan’s BGISEQ-50 platform. The classification reference databases were downloaded from the National Center of Biotechnology Information (ftp://ftp.ncbi.nlm.nih.gov/genomes), whose RefSeq database contains 4,189 whole-genome sequences of viral taxa, 2,328 bacterial genomes or scaffolds, 199 fungi related to human infection, and 135 parasites associated with human diseases.

**Figure 2:**
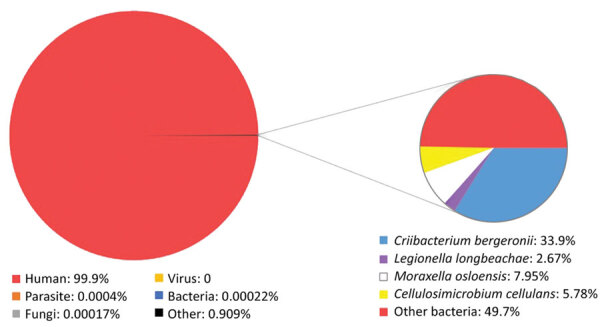
Analysis of metagenomic next-generation sequencing result from a patient with *Legionella longbeachae*. Total reads distribution is on the left; percentage distribution of bacterial reads is shown on the right.

On the patient’s 14th day in the hospital, the results of mNGS were positive for *L. longbeachae* (Figure 2). We began treatment with intravenous moxifloxacin (400 mg/d) and oral azithromycin (500 mg/d); meropenem was withdrawn. The patient was afebrile and his cough diminished.

On his 25th day in the hospital, the patient had an epileptic seizure. The CT of skull and chest revealed that the brain was normal, and the lungs had a smaller consolidation than before (Figure 1, panel B). After considering the side effects of moxifloxacin, we discontinued treatment. The seizure did not recur. Six days later, the patient developed another cough with a low-grade fever. We performed a new BAL and cultures were still negative. We started treatment again with 400 mg of intravenous moxifloxacin daily, and the patient’s symptoms improved.

On his 36th day in the hospital, the patient had another seizure. Intravenous moxifloxacin was discontinued again and 400 mg oral moxifloxacin daily was started. An electrocardiogram (ECG) revealed a prolonged corrected QT interval. After considering the cardiologic side effects of azithromycin, we withdrew the azithromycin. However, the patient experienced a fever again and the sputum cultures showed no growth. The epilepsy did not improve despite pumping depakin continuously.

 On the patient’s 39th day in the hospital, we began treatment with intravenous ciprofloxacin and tigecycline because of his worsening condition. The following day, he had a temperature of <41°C. Oxygen saturation was 93% while he was on 28% fraction of inspired oxygen. That night, the patient suffered cardiac arrest and died. The patient’s family refused to have an autopsy performed.

Since *L. longbeachae* was first identified as a new species in 1981 (*1*), legionellosis caused by *L. longbeachae* has been reported in many countries, including Australia, New Zealand, Netherlands, Japan, and Canada (*2**–**6*). However, to our knowledge, there are no relevant reports from countries such as mainland China, Russia, South Korea, and India. Some reasons may explain the discrepancy. On one hand, *L. longbeachae* is commonly isolated from compost and potting mixes (*7*). The composition of commercial potting mix used in different countries is different (*8*). On the other hand, the PCR technique for *L. longbeachae* is immature in many regions, so the urinary antigen test is used, which detects only *L. pneumophila* serogroup 1 (*9*). Even in countries with low incidence rates for *L. longbeachae* infection, when the cultures and urine antigen test are negative, a high index of suspicion must be maintained for patients with immunosuppression and a personal history of gardening activities.

Detection by culture techniques is insensitive for *Legionella* spp. (*10*). At present, in some countries with high incidence of *L. longbeachae* infection, such as Australia and New Zealand, PCR is the most commonly used tool to detect *Legionella* spp. (*8**,**9*). In most areas of China, PCR technique for *L. longbeachae* is still immature. However, mNGS is rapidly moving from research to clinical practice (*11*). As of November 2019, >100 case reports or clinical studies have indicated that mNGS has been successfully applied in dozens of sample types, such as respiratory secretions, cerebrospinal fluid, urine, and blood (*12*), but it is rarely reported that *L. longbeachae* infection is diagnosed by mNGS. In this case, using a novel mNGS platform, we were able to detect *L. longbeachae* in a BALF sample and finally confirm the etiology of the patient’s pneumonia. The result indicated that mNGS may be a useful tool for detecting *Legionella* spp.

Antimicrobial susceptibility testing of *L. longbeachae* has demonstrated that quinolones, macrolides, and rifampin were most sensitive to it (*13*). However, there are few relevant reports of developing side effects after using quinolones for patients with *L. longbeachae* infection. Unfortunately, our patient not only had a seizure after using moxifloxacin but also had a prolonged QT interval at the later stage of therapy. Therefore, the available antimicrobial drugs were very limited. Looking back to the therapy process of the patient, our treatment regimen had some deficiencies. First, considering that other quinolones may also cause seizure, we did not switch to other types of quinolones, and ciprofloxacin was used until the day before the patient died. Second, considering that other types of macrolide drugs were not as effective as azithromycin and also had the possibility of prolonging the QT interval, dosage of the macrolide drugs had not been adjusted. We possibly should have switched to intravenous erythromycin while closely monitoring ECG changes. Third, given the relatively weak efficacy of rifampin, we did not try to add rifampin. In fact, rifampin could have been added when seizures were repeatedly caused by moxifloxacin. However, the patient died of sudden cardiac arrest and ECG was not performed in time. The cause of cardiac arrest may have been that ciprofloxacin or deteriorating pneumonia aggravated the patient’s arrhythmia. More treatment experience and reports are needed for such patients.

## Conclusions

In summary, even in countries with low incidence rates for *L. longbeachae* infection, when the cultures and urine antigen test are negative, *L. longbeachae* infection must be highly suspected for patients with immunosuppression and a personal history of gardening activities. This case indicated that mNGS may be a useful tool to diagnose *L. longbeachae* infection. For patients with *L. longbeachae* infection, antimicrobial drugs should be changed in time when patients develop adverse side effects after using moxifloxacin and azithromycin; more treatment experience and reports are needed for such patients.
